# Supplementary material for: pycoMeth: a toolbox for differential methylation testing from Nanopore methylation calls
Source: Genome Biol. 2023 Apr 20;24:83. doi: 10.1186/s13059-023-02917-w (PMC10120131; doi:10.1186/s13059-023-02917-w)
Supplement: Supplementary file 1 — Additional file 1: Supplementary figures S1-S11. [file 13059_2023_2917_MOESM1_ESM.pdf]

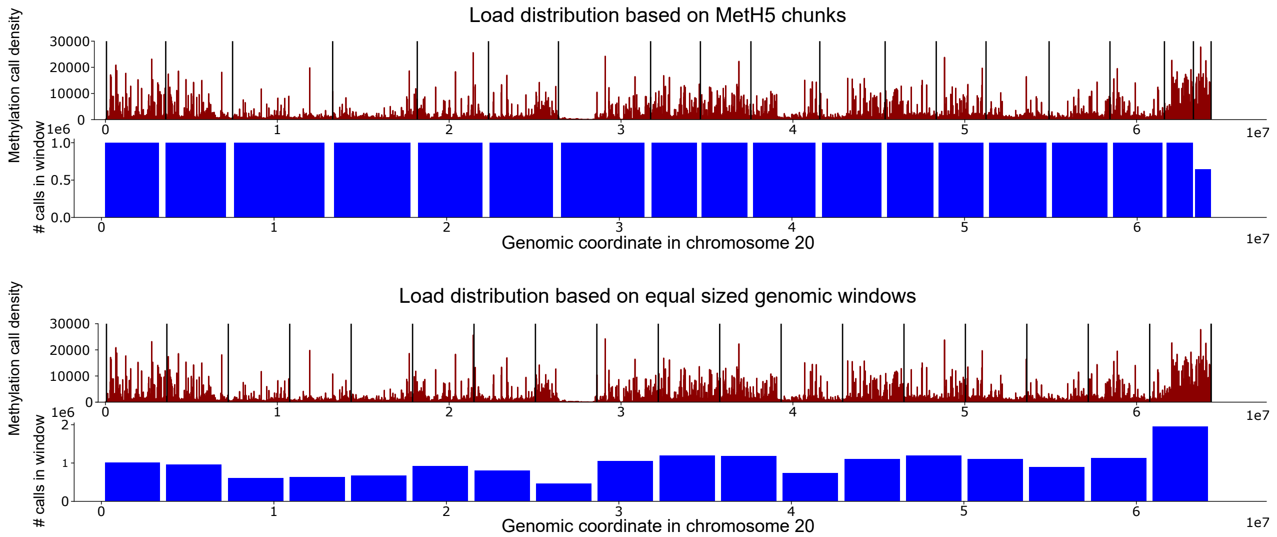

Fig. S1: Parallel computing first requires splitting the input dataset for each parallel task. Good load distribution requires even distribution of input data. A naïve approach can be to distribute load based on evenly spaced genomic windows. However, CpG density and coverage are not even across an entire chromosome, leading to an uneven distribution of methylation calls per parallel task. MetH5 stores methylation calls in a chunked fashion. Definining parallel operations over MetH5 chunks results in an even load distribution across all parallel tasks. Methylation call density plotted here is computed as the total number (from all mapped reads) of methylation calls in a 1000bp window on HG002.

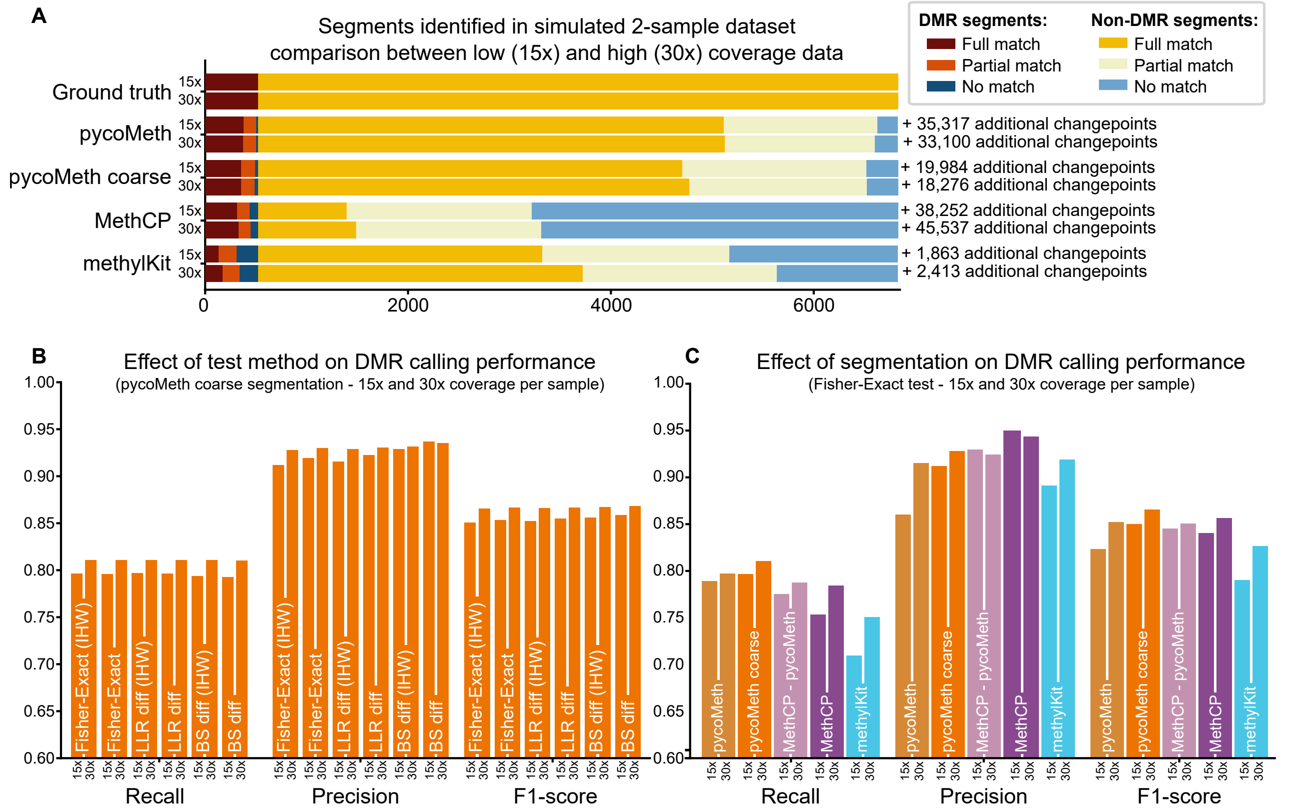

Fig. S2: Benchmark on a simulated 2-sample dataset with lower coverage (15x). A) Compares the metrics analyzed in Figure 4A for 30x coverage simulated data with a lower coverage simulation of the same methylation profile at 15x. PycoMeth segmentations are largely unaffected, while MethCP and methylKit segmentations suffer from the drop in coverage. B) and C) show DMR calling recall, precision and F1-score for low coverage simulated data, analogous to Figure 4B-C, where each bar group corresponds to the respective interpretation of the y-axis: recall as a measure of test power, precision as a measure of false discovery, and F1-score (harmonic mean of recall and precision).

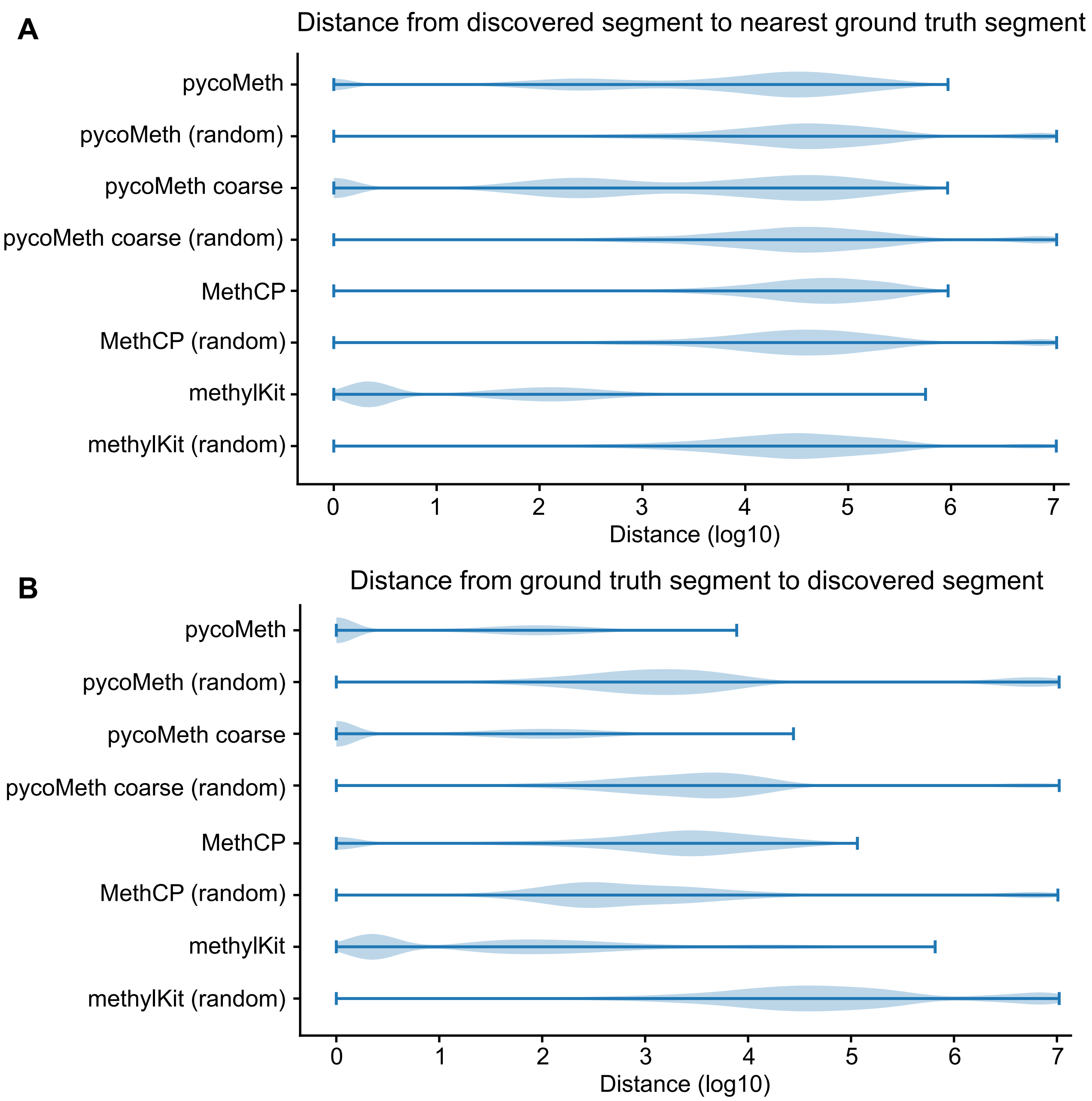

Fig. S3: Permutation test on segmentations. In a permuted segmentation the original predicted segments retain their sizes but are shuffled in their order. This simulates a random segmentation with the same granularity. **A**) Distance from a discovered (predicted) changepoint to the nearest ground-truth changepoint. **B**) Distance from each ground-truth changepoint to the nearest predicted changepoint.

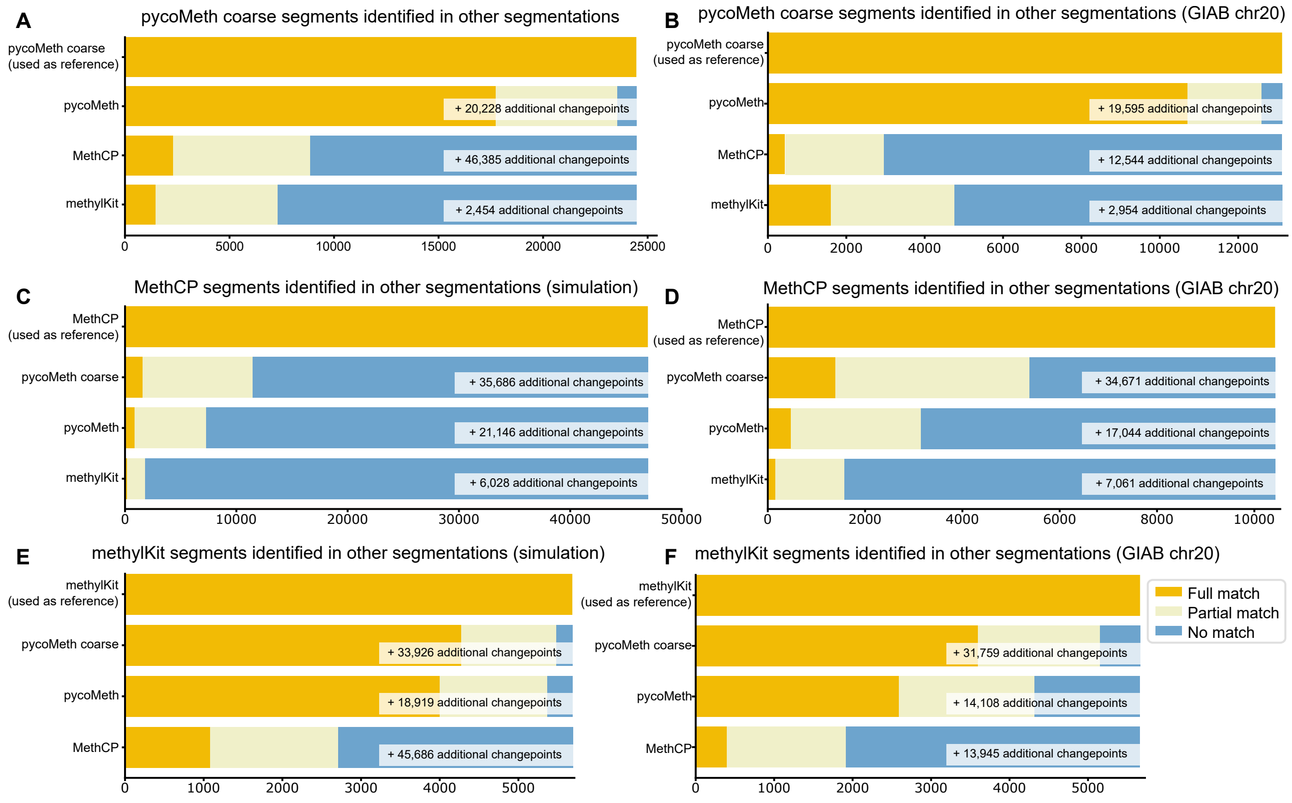

Fig. S4: Agreements between segmentations, setting one segmentation as reference and visualizing how many of the reference segments can be identified by other segmentations. **Full match**: both sides of a segment have been found. **Partial match**: one side of the segment has been found. **No match**: neither side of the segment has been found. **A-B** use pycoMeth coarse segmentation as a reference in simulated and GIAB parent comparison, respectively. **C-D** use MethCP as a reference and **E-F** use methylKit as a reference.

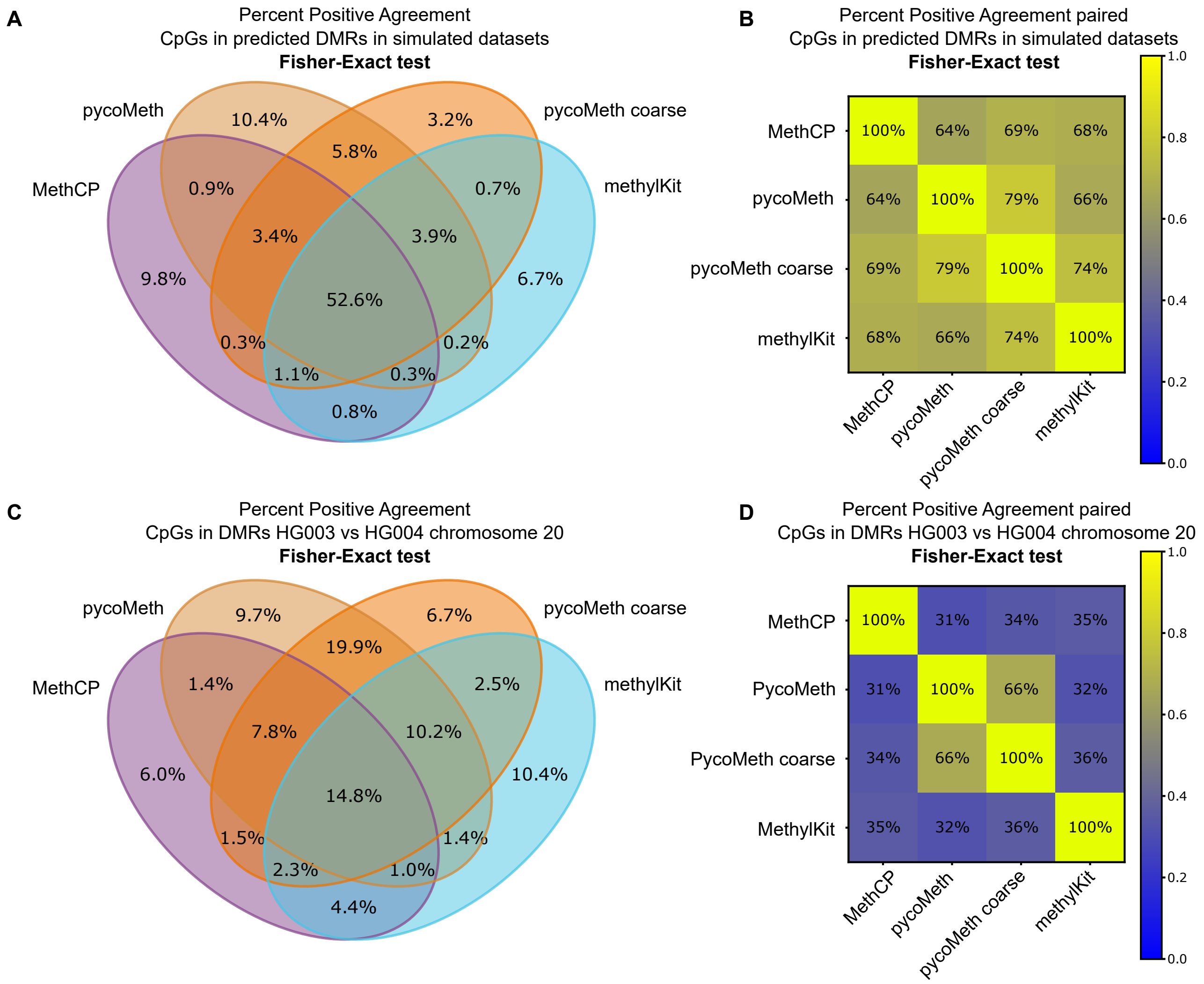

Fig. S5: Percent positive agreement of different segmentations measured as intersection of CpGs in DMRs called in the simulated data (**A-B**) and on chromosome 20 in the GIAB data parent comparison (**C-D**). Segmentation on the simulated data was overall more consistent, potentially due to the more homogeneous distribution of effect sizes. Agreement between pycoMeth coarse and pycoMeth segmentations is 79.3% in the simulated analysis and 66.5% in the GIAB parent analysis.

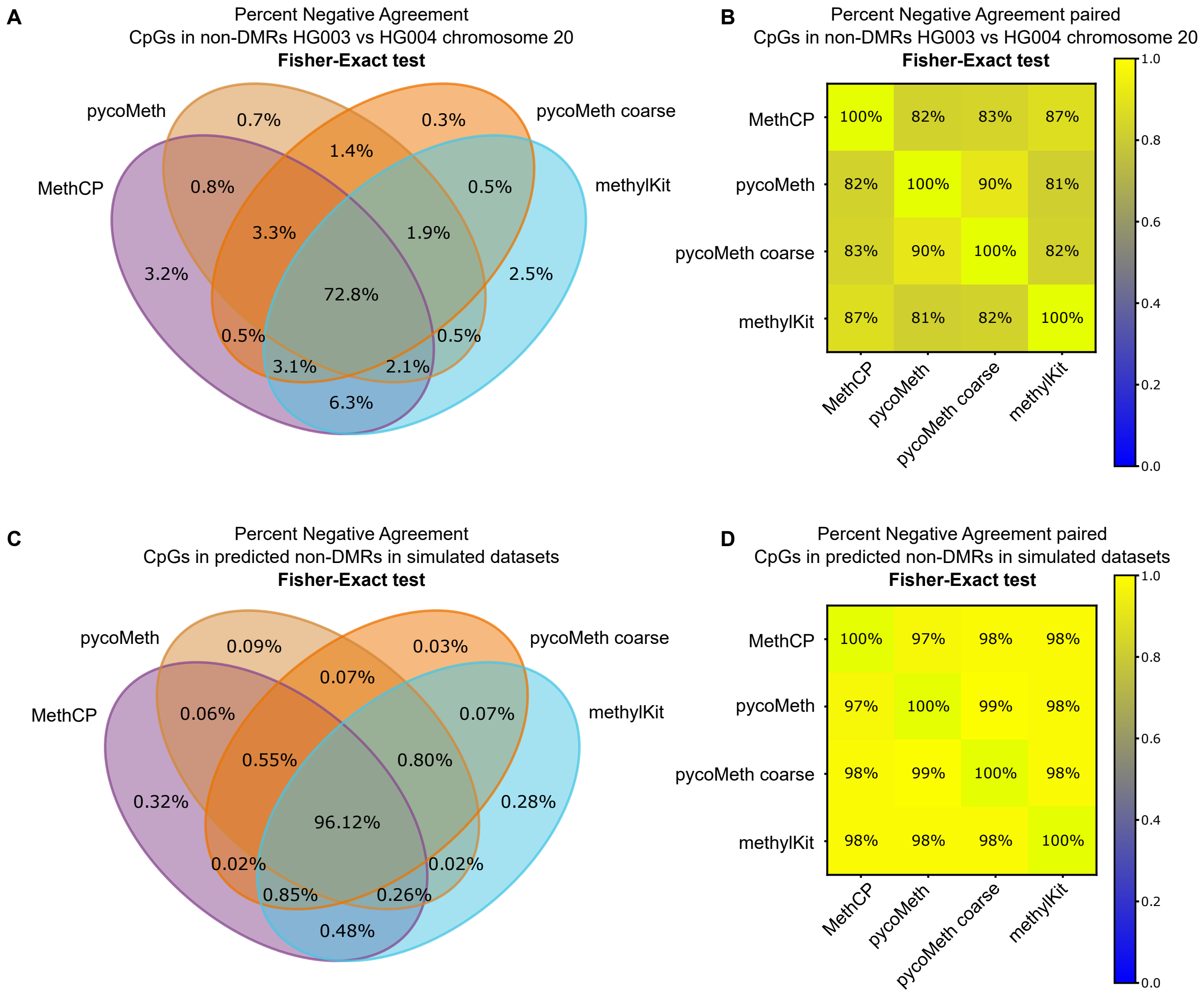

Fig. S6: Percent negative agreement of segmentations measured as intersection of CpGs not in DMRs called in the simulated data (**A-B**) and on chromosome 20 in the GIAB data parent comparison (**C-D**).

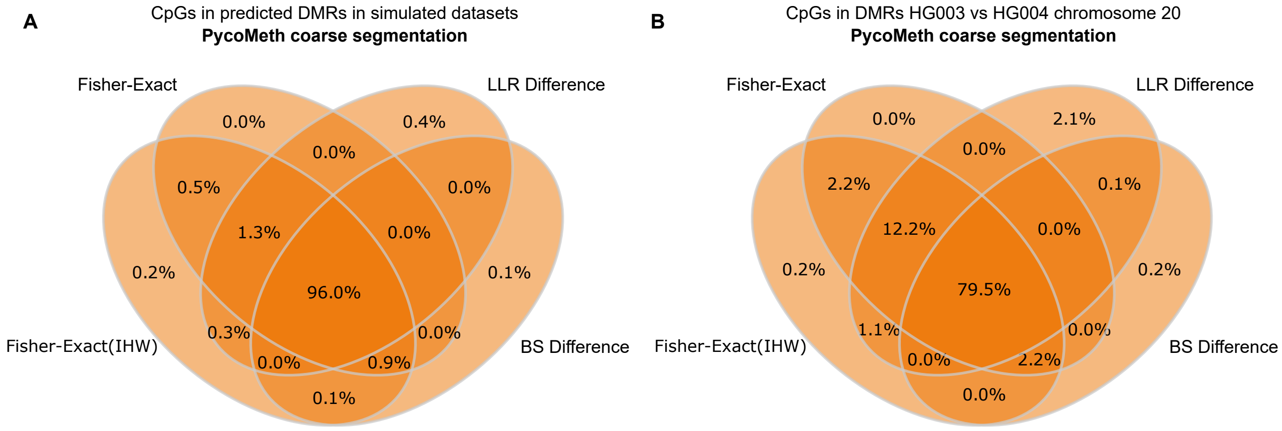

Fig. S7: Percent positive agreement of different tests measured as intersection of CpGs in DMRs called in the simulated data (**A**) and on chromosome 20 in the GIAB data parent comparison (**B**).

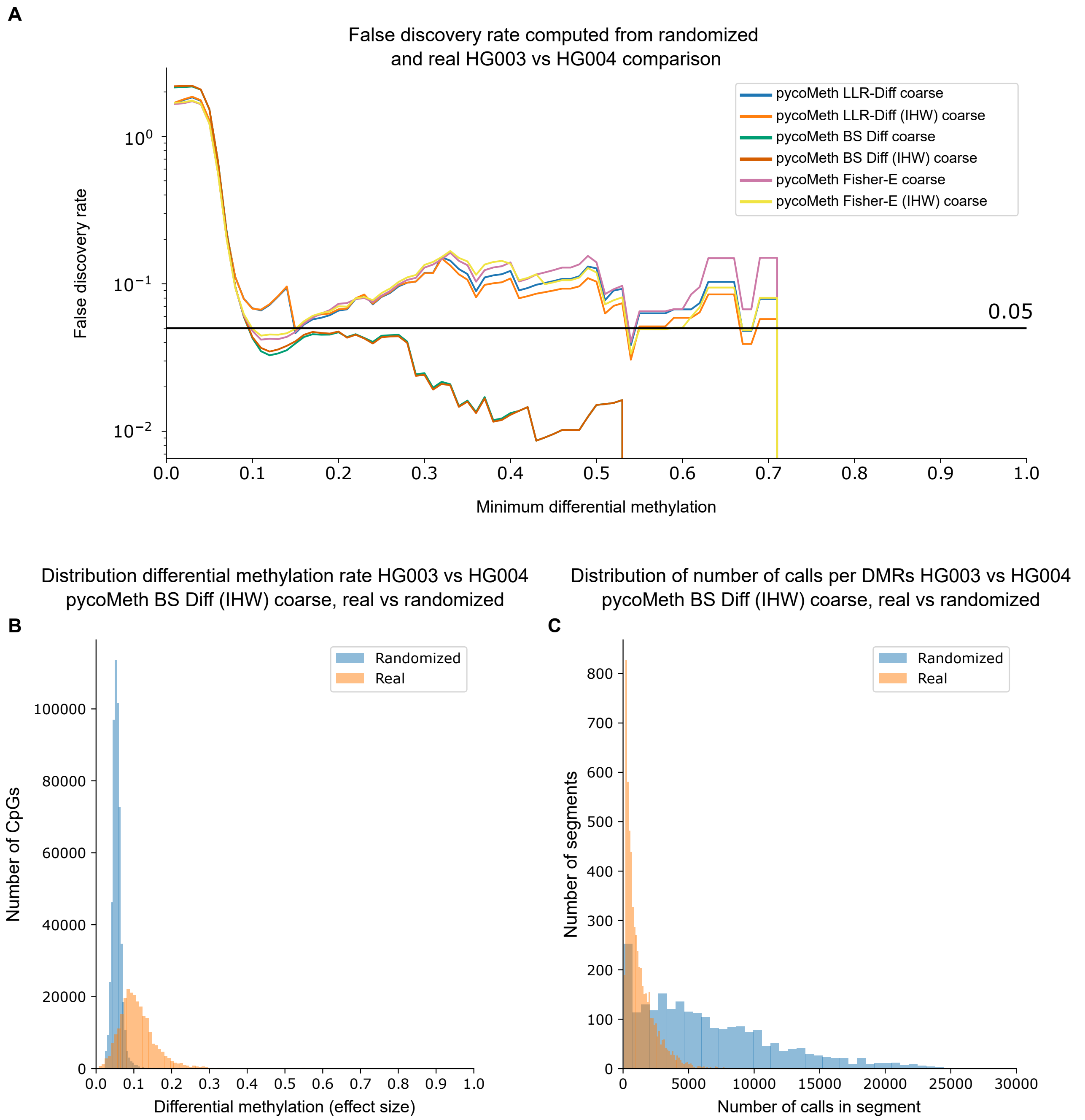

Fig. S8: Randomization test results for comparison between HG003 and HG004. For the randomized dataset, for each sample and each chromosome LLRs have been shuffled to remove any read or site-dependent information. **A)** False discovery rate computed as the number of CpGs in DMRs in the randomized dataset divided by the same number for the real HG003 and HG004 comparison, plotted over the minimum segment differential methylation rate. We observe high FDR in segments with less than 0.1 differential methylation. The most conservative test implemented in pycoMeth (BS Diff) shows best FDR overall, and IHW appears to slightly reduce FDR in general. **B** Distribution of segment differential methylation (represented by CpGs in the segment) in called DMRs between the real and randomized dataset, from pycoMeth coarse with BS Diff test hypothesis and IHW. **C)** Distribution of calls per segment in called DMRs between the real and randomized dataset, from pycoMeth coarse with BS Diff test hypothesis and IHW.

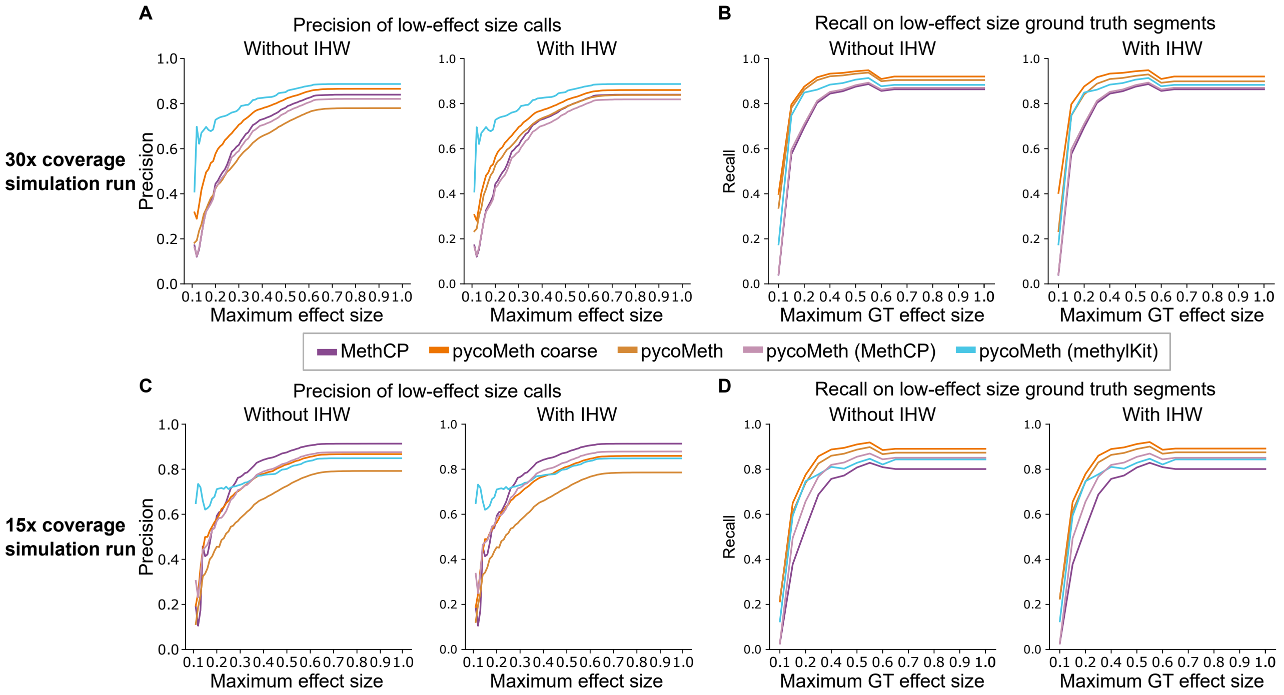

Fig. S9: Investigating over-calling of low-effect size methylation calls. **A-B**) Precision and recall for effect-size capped DMR predictions with and without IHW on the high coverage simulation example. **C-D**) Matching analysis on the low coverage simulation example.

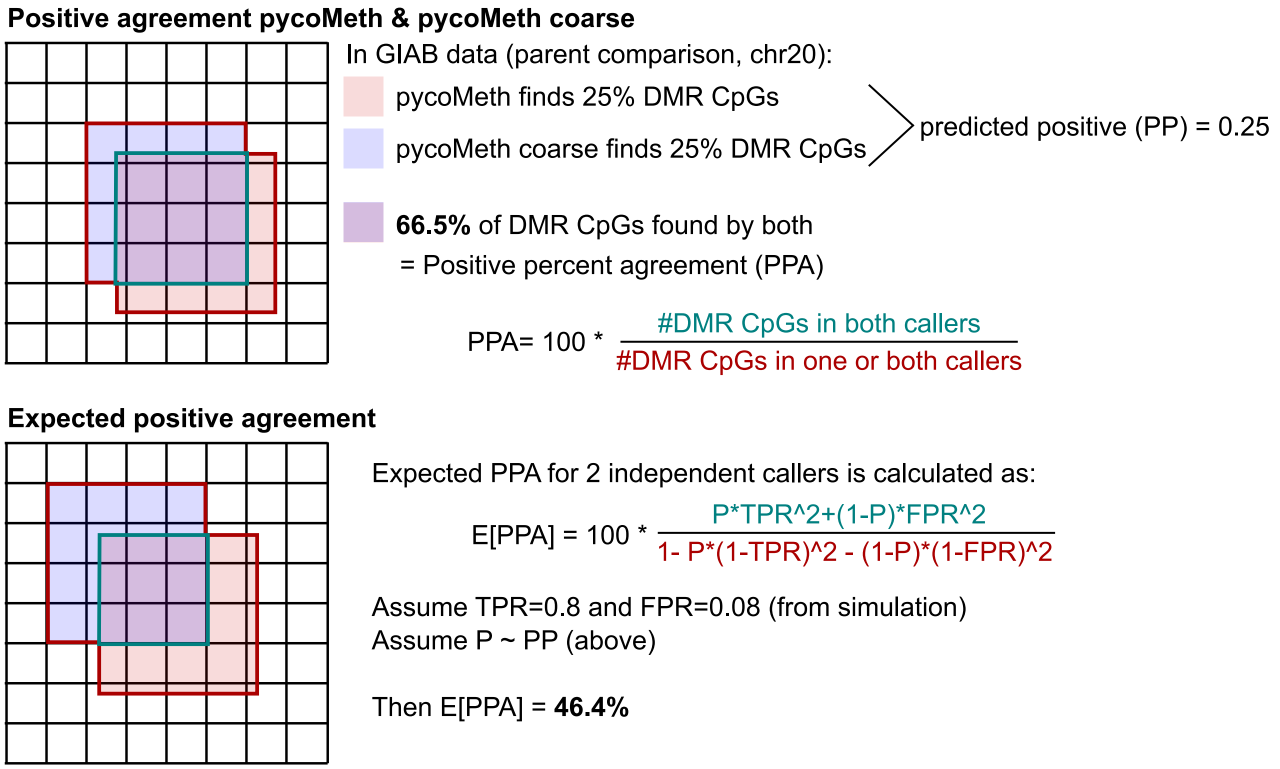

Fig. S10: Illustrating how percent positive agreement is computed and how it relates to distribution of labels (DMR CpG versus non DMR CpG), as well as how expected PPA is computed for evaluation of DMR calling consistency. With the true positive rate (TPR) and false positive rate (FPR) estimated from the simulation benchmark, and assuming 25% DMR CpGs as predicted by pycoMeth, the expected PPA would be 46.4%. Comparing both pycoMeth segmentations yields a PPA of 66.5%, showing good consistency between DMRs called on both segmentations.

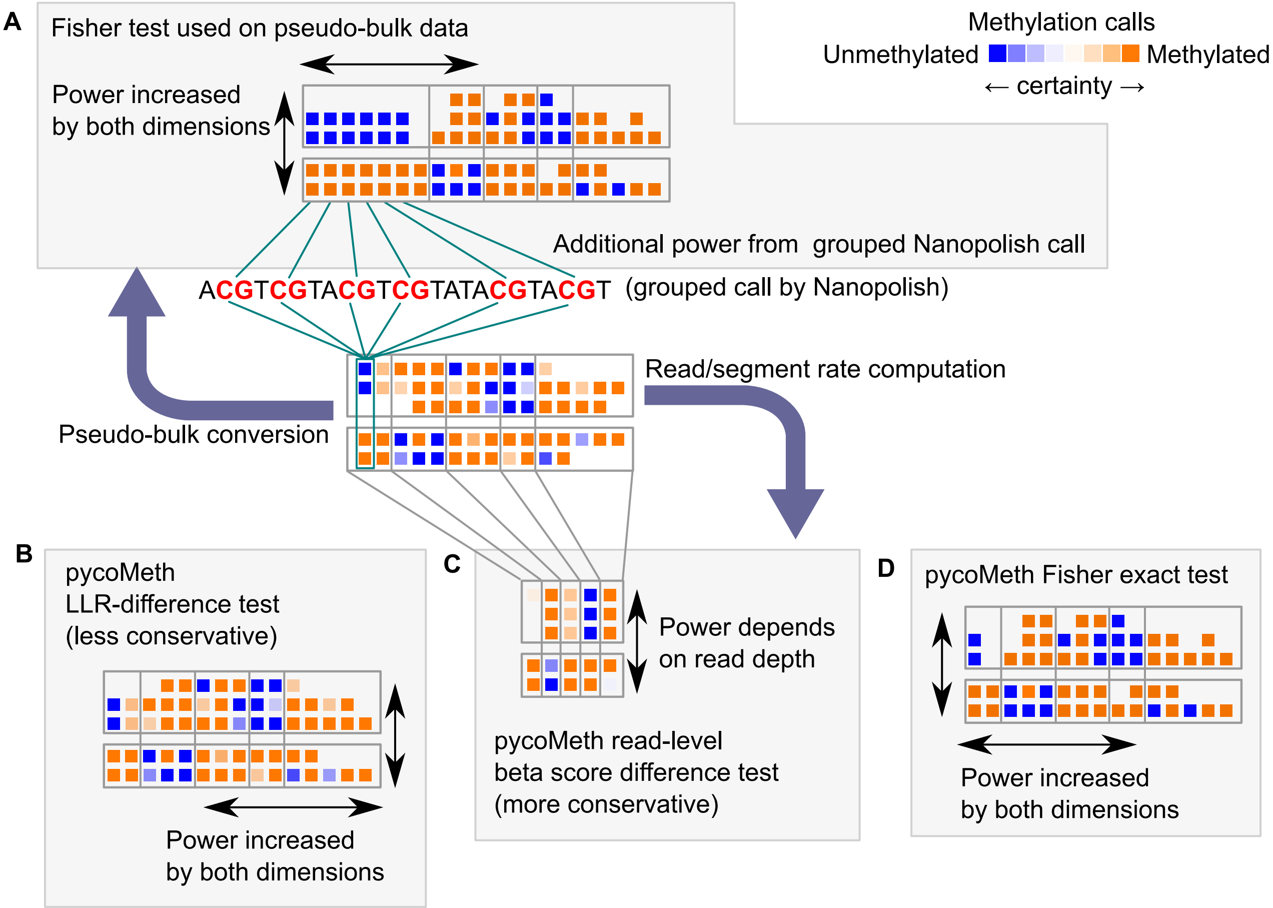

Fig. S11: Illustration of where different differential methylation testing methods draw their power. **A**) In attempting to analyze Nanopolish methylation calls with tools developed for bulk bisulfite sequencing data, we create pseudo-bulk data. Tests generated for pseudo-bulk comparison (such as MethCP which we evaluated in this work) test based on CpG-level methylation rates and coverage across all reads and therefore draw power from the segment size and read depth. Furthermore, since Nanopolish generates grouped calls for nearby CpG-sites, some calls are therefore not independent and thus artificially generate more testing power. **B**) PycoMeth with the parameter "–hypothesis llr_diff" performs a less conservative test implemented in the pycoMeth package, where each individual methylation call is treated as independent and samples are compared based on their LLR distribution. Here discovery power is determined also by a combination of segment length and sequencing depth. **C**) PycoMeth with the parameter "–hypothesis bs_diff" instead computes a methylation rate per read per segment and draws power only from the independent information (sequencing depth). **D**) Fisher Exact test as implemented in pycoMeth for two-sample tests with the parameter "–hypothesis count_dependency".
