# Supplementary material for: pycoMeth: a toolbox for differential methylation testing from Nanopore methylation calls
Source: Genome Biol. 2023 Apr 20;24:83. doi: 10.1186/s13059-023-02917-w (PMC10120131; doi:10.1186/s13059-023-02917-w)
Supplement: Supplementary file 3 — Additional file 3. Review history. [file 13059_2023_2917_MOESM3_ESM.docx]

Review History

**First round of review**

**Reviewer 1**

**Were you able to assess all statistics in the manuscript, including the appropriateness of statistical tests used?**

Yes, and I have assessed the statistics in my report.

**Were you able to directly test the methods?**

Yes.

**Comments to author:**

-Is the Software likely to be of broad utility? Is it easy to install and use?

The authors describe two software solutions to be used for downstream analysis of methylation calling data detected by Nanopolish, a well-established software package for signal-level analysis of Oxford Nanopore Technologies (ONT) sequencing data. MetH5 implements an HDF5-based container format for efficient storage and retrieval of methylation calls from long reads. The pycoMeth software provides tools for the most common use-cases when dealing with methylation data, namely the segmentation of methylation profiles, testing for differential methylation and generation of result files in BED format together with comprehensive HTML reports.

The software can be easily installed using the package installer for Python (pip) or the conda package manager, and can conveniently be accessed via Python API or via a command-line interface.

-Does the software represent a significant advance over previously published tools (as demonstrated by direct comparison with available related software)?

For work with long read methylation calls, the MetH5 container provides a strong improvement in terms of file size and access speed over conventional CRAM or BAM file storage. The segmentation algorithm implemented in the pycoMeth suite is able to simultaneously detect the majority of differentially methylated regions (DMRs) and segments with similar methylation signals (Non-DMRs), whereas segmentation using existing solutions methylKit and methCP only captures either most Non-DMRs or DMRs, respectively.

-Please indicate briefly the novel features and/or advantages of the software and/or please reference the relevant publications and which alternatives, if any, it should be compared with.

The authors introduce a new storage solution for methylation data as generated by nanopore sequencing, based on the broadly adopted hierarchical data format in Version 5 (HDF5), which is already used to store the raw data produced by the ONT sequencers. Thereby, the authors were able to reduce the storage requirements for the methylation calls compared to existing solutions and at the same time increase the speed to access the data.

The authors provide a software suite to perform common analysis from methylation data, namely methylome segmentation and differential methylation testing. The novelty here is, that the authors developed new methods incorporating the uncertainty of each single methylation call provided as log-likelihood-ratios (LLRs) of methylation. By employing the methylation call uncertainties, it is possible to mitigate choosing rigorous coverage thresholds as required in traditional short-read sequencing approaches. The idea to include methylation call uncertainty as prior information for modelling and testing is also a great approach to recovering sparsely covered CpGs in low-input samples.

For segmentation of the methylome, the authors implement a Bayesian changepoint detection algorithm model as Hidden Markov Model (HMM). This has some major benefits over existing tools using fixed-state HMMs for methylome segmentation, like MethylSeekR [1], since the number of states will be determined based on the given signal. To the knowledge of the reviewer, this is a novel approach, given that competing tools like MethCP [2], methylKit [3] or metilene [4] mostly resort to variations of the circular binary segmentation [5] for change point detection.

-Are the methods appropriate to the aims of the study, are they well described, and are necessary controls included?

The methods are well-described and appropriate for this type of comparison.

-Are the conclusions adequately supported by the data shown?

Yes.

-Are sufficient details provided to allow replication and comparison with related analyses that may have been performed?

The data and methods are comprehensively explained to recapitulate what has been done.

There are just a few minor points that need to be addressed before publication: In the methods section, some tools are missing the used software versions: minimap2 (page 16, line 46), samtools (page 16, line 58), methylKit (page 22, line 9) and MethCP (page 22, line 9). On page 16 line 62 the meth5 API version 0.8.0 is mentioned, but there is no release associated with this version, neither on GitHub nor on PyPI. The files in the main/master branch of the GitHub repository for the benchmark scripts are not up to date. The website (https://a-slide.github.io/pycoMeth/) linked from the pycoMeth GitHub repository is out of date.

-Is the paper of broad interest to others in the field, or of outstanding interest to a broad audience of biologists?

The paper is of interest to others in the field, looking for an end-to-end solution to analyze CpG-methylation in nanopore sequencing data.

[1] Lukas Burger, Dimos Gaidatzis, Dirk Schübeler, Michael B. Stadler, Identification of active regulatory regions from DNA methylation data, Nucleic Acids Research, Volume 41, Issue 16, 1 September 2013

[2] Boying Gong and Elizabeth Purdom.MethCP: Differentially Methylated Region Detection with Change Point Models.Journal of Computational Biology.Apr 2020.458-471.

[3] WRECZYCKA, Katarzyna, et al. Strategies for analyzing bisulfite sequencing data. Journal of biotechnology, 2017, 261. Jg., S. 105-115.

[4] JÜHLING, Frank, et al. Metilene: fast and sensitive calling of differentially methylated regions from bisulfite sequencing data. Genome research, 2016, 26. Jg., Nr. 2, S. 256-262.

[5] Adam B. Olshen, E. S. Venkatraman, Robert Lucito, Michael Wigler, Circular binary segmentation for the analysis of array‐based DNA copy number data, Biostatistics, Volume 5, Issue 4, October 2004

**Reviewer 2**

**Were you able to assess all statistics in the manuscript, including the appropriateness of statistical tests used?**

No, I do not feel adequately qualified to assess the statistics.

**Were you able to directly test the methods?**

No.

**Comments to author:**

Snajder et al. provide a tool for differential methylation analysis and propose a complementary data storage format for ONT reads. In this report the authors introduce the pycoMeth toolbox to store, manage and analyse DNA methylation data obtained from long-read ONT sequencing data. The toolbox is built around a new file format 'MetH5' proposed as a complementary format to the BAM standard, that allows efficient storage of and rapid data access for read-level and reference-anchored methylation call data. In addition to support for standard read level operations on MetH5 file format, pycoMeth provides a series of differential methylation test options through the Meth_Seg and Meth_Comp modules. These include a Bayesian changepoint detection framework as a methylome segmentation tool and DMR reporting capabilities across a range of sample numbers. pycoMeth is benchmarked against simulated and real data using MethCP and methylkit as comparative platforms.

Synopsis: This report addresses a gap in the field providing a framework for improved file management and differential methylation analysis capabilities for ONT datasets. Heuristic trade-offs in the proposed MetH5 file type, increased speed for larger size, and the interoperability of the BAM file type (compared to MetH5) may limit uptake. The overall performance of pycoMeth on simulated data and against existing tools highlights the need for additional development. Thus while this study does not appear to provide an optimal solution to DMR analysis using ONT datasets it does provide a well-reasoned exploration of the utility of the Bayesian methylome segmentation approach.

Critiques:

1. MetH5 provides for faster processing times but at the expense of file size and thus its utility may be limited as a primary storage filetype. Perhaps it would be useful file type during the analysis phase with the option to output as a BAM/CRAM file for final storage.

2. Overall performance of the pycoMeth (both normal and coarse) is not particularly compelling with ~50% agreement even between pycoMeth and pycoMeth coarse. Given this, the authors provide suggested parameters for running Meth_Comp, but it is clear that even this is not an optimal solution. The description of the benchmarking provided on page 11 is difficult to follow "Looking again at chromosome 20 and comparing with the tests on simulated data, only 14.8% of CpGs in DMRs found in the parent comparison are found by all segmentations, whereas in the simulated case, the agreement was 52.6%." - is the former the real data and the later the simulated dataset?

3. Figures are difficult to interpret with colours and labels not fully described. For example in figure 1 (and elsewhere), what do the orange and blue boxes indicate, in Figure 4A, what does 'no match' or 'partial match' indicate in this context - does it mean that for no DMR regions, the tool called as a DMR as a 'no match'? Some figure labels are unreadable or cut off (e.g. Figure 4B, C).

4. Would the authors comment on the utility of running MethylSeg/Comp on its own - or would they argue for a METEORE like approach combining calling platforms together?

**Reviewer 3**

**Were you able to assess all statistics in the manuscript, including the appropriateness of statistical tests used?**

Yes, and I have assessed the statistics in my report.

**Were you able to directly test the methods?**

Yes.

**Comments to author:**

Snajder et al. present a toolkit for methylation analysis. The manuscript is written well, and I was able to follow the text as well as the methods and results. I think this is a useful tool and am in favor of the manuscript in general. I have a few minor questions:

1. The authors mentioned that the toolkit should work for GpC as well as 6mA modifications. Could they comment on the possibility of examining 5mC and 5hmC on the same CpG motifs? Is that doable in the existing implementation or will it require code development?

2. I appreciate the complexity and challenges associated with various file formats and incompatibilities. What is the roadmap for integrating with modBAM formats? Tools like modbamtools are handling the methylation tags now directly from Guppy basecalls which is quite nice. I think there would be an even higher adoption for pycoMeth (and possibly MetH5) with integration for that perhaps?

Good luck to the authors!

**Authors Response**

**Point-by-point responses to the reviewers’ comments:**

Reviewer #1

* R1.1: In the methods section, some tools are missing the used software versions: minimap2 (page 16, line 46), samtools (page 16, line 58), methylKit (page 22, line 9) and MethCP (page 22, line 9).

*Reply: We thank the reviewer for pointing out the missing software versions, we have now included the complete version numbers in the text.*

*Specifically:*

*“Alignment to reference genome GRCh38 was performed using minimap2 (version 2.17) [26]” (page 15, line 25)*

*“BAM files were compressed to create CRAM files using samtools (version 1.5) [15]” (page 15, line 32)*

*“The pycometh methylome segmentation was compared to two existing tools designed for WGBS, methylKit (version 1.18.0) [19] and MethCP (version 1.6.0) [20]” (page 21, line 21)*

* R1.2: On page 16 line 62 the meth5 API version 0.8.0 is mentioned, but there is no release associated with this version, neither on GitHub nor on PyPI.

*Reply: We appreciate this comment and have created the appropriate tag and release on GitHub.*

*See: https://github.com/PMBio/MetH5Format/releases/tag/v0.8.0*

* R1.3: The files in the main/master branch of the GitHub repository for the benchmark scripts are not up to date.

*Reply: The additional benchmarks done between the first preprint and the journal submission were in a different branch. We have now merged this into the main branch, and made a release corresponding to this version. We thank the reviewer for the time to look into the software and pointing out the inconsistencies.*

* R1.4: The website (https://a-slide.github.io/pycoMeth/) linked from the pycoMeth GitHub repository is out of date.

*Reply: To address this we have updated the documentation for changes made in pycoMeth version 2, which is now available under https://pmbio.github.io/pycoMeth and is also linked from the pycoMeth GitHub repository. In order to avoid confusion, we also unforked the canonical GitHub repository from the original (now deprecated) pycoMeth version 1 repository.*

Reviewer #2

* R2.1: MetH5 provides for faster processing times but at the expense of file size and thus its utility may be limited as a primary storage filetype. Perhaps it would be useful file type during the analysis phase with the option to output as a BAM/CRAM file for final storage.

*Reply: We thank the reviewer for this comment and agree that MetH5 is mostly a file format that is very well suited for analysis as it allows for rapid access times and low memory usage data access. The latest releases of ONT DNA-methylation callers, such as guppy, remora, and the newer versions of Nanopolish, have already moved to writing BAM files containing methylation information. We therefore expect that in the future, methylation storage in BAM files will be the initial format and also used for long-term archiving.*

*However, for analysis, as also pointed out by the reviewer we feel this is still a very useful format. Additionally for sharing DNA-methylation information, without sharing donor genotypes, and focused analysis on DNA-methylation we feel this is still a very useful file format.*

*When performing analysis or visualization of methylation data, we find that conversion to MetH5 followed by analysis using data stored in MetH5 vastly outperforms performing analysis directly on BAM files. Furthermore, since methylation data is but a fraction of the total BAM file (in our benchmark 2-3%), one may even decide to archive both the BAM file and the MetH5 file in order to boost future methylation analyses. We have addressed this in our discussion, but in light of the reviewer’s comments we attempted to make it a bit more explicit in this revision.*

*Specifically the relevant section in the discussion now reads:*

*”While we are aware of the advantages of having methylation calls stored in a well-supported file format, we believe MetH5 to be a complementary solution used during analysis and visualization, offering boosted performance, parallel computing capabilities and an intuitive interface”*

*Additionally to future proof our code base we integrated a conversion subcommand in the MetH5 CLI, which allows for convenient conversion from modBAM to MetH5. However, we are currently blocked by upstream dependency issues. The MetH5 version currently in the “dev” branch (release v1.2.0_alpha) on github contains a subcommand “convert” which allows for efficient BAM -> MetH5 conversion. However, the canonical library for reading BAM files in python, pysam (which itself wraps HTSLib), has not yet been adapted to reading the MM tags written by guppy, remora, and Nanopolish correctly. HTSlib version 1.16 which was released in August 2022 implemented compatibility, yet pysam has not yet been updated. Once pysam issue #1123 (https://github.com/pysam-developers/pysam/issues/1123) has been fixed, we are ready to immediately publish MetH5 version 1.2.0 which includes the conversion subcommand.*

* R2.2: Overall performance of the pycoMeth (both normal and coarse) is not particularly compelling with ~50% agreement even between pycoMeth and pycoMeth coarse. Given this, the authors provide suggested parameters for running Meth_Comp, but it is clear that even this is not an optimal solution.

*Reply: We thank the reviewer for this comment. While we agree that at this point it is difficult to refer to any proposed solution as an optimal solution, we would like to clarify the statistics presented here.*

*The agreement cited here is the positive percent agreement (PPA), which in an unbalanced dataset with fewer positive labels than negative is inherently lower. Upon closer inspection we realize that the 52.7% written in the manuscript may have been misleading, since it refers to the agreement relative to the union of all four callers. When comparing the two methods pycoMeth and pycoMeth coarse, however, we should compute PPA relative to only the union of only these two callers. We have updated these numbers in the text to 66.5% agreement between the two pycoMeth segmentations in the GIAB parent analysis and 79.3% agreement in the simulated data (these numbers can also be found in updated Supplementary Figure 6).*

*To put these numbers in perspective, given the estimated true positive rate of ~0.8 and a false positive rate of ~0.08 (as derived from our benchmark on simulated data). Assuming the incidence of positives in the ground truth matches the predicted incidence by pycoMeth (roughly 25% in the GIAB parent comparison chromosome 20) the expected PPA of two independent predictions with these accuracies would be roughly 46.4% (compared to the observed 66.5%). With an incidence of only 8% as in our simulated data, the expected PPA would be 26.2% (compared to the observed 79.3%).*

*In the results, we mainly intended to point out PPA as a comparison point between real and simulated data, in order to show that there are factors which do make the problem somewhat more complex in real data. Still, the presented PPA on the real data is in an expected range. We have better illustrated PPA and percent negative agreement (PNA), also with pairwise comparisons, in the new Supplementary Figures 7-8.*

*Furthermore, in order to show the consistency of segmentation, we have analyzed segmentation consistency by taking one segmentation as reference and analyzing segment detection using other segmentations, analogously to our segmentation benchmark where ground truth segmentation was used as a reference. There, we find that pycoMeth and pycoMeth course largely agree, with 84% of changepoints identified by pycoMeth also identified by pycoMerh coarse, while pycoMeth as the more fine-grained segmentation further breaks down the same segments. This is illustrated in the new Supplementary Figure 6.*

*We have rewritten the relevant paragraphs to make this more clear and this now reads:*

*”Surprisingly we find that the tool used for segmentation has a great impact on the segment discovery, implying that the methods are complementary. High agreement in the segmentation performed by pycoMeth coarse and pycoMeth show that increasing segmentation granularity in pycoMeth still finds most of the same changepoints, but increases how segments are further broken down (Supplementary Figure 6).*

*Comparing agreement of differential methylation calling, we find that in the real data (GIAB parent comparison, chromosome 20) only 14.8% of CpGs in DMRs identified are found using all segmentations, much lower than the 52.6% observed on the simulated data. Between pycoMeth and pycoMeth coarse, the percent positive agreement (PPA, Methods) in the real data was 66.5% (79.3% in simulated data, Supplementary Figure 7). Note that the PPA of 66.5% is higher than expected (46.4%) at the observed DMR distribution (Methods).*

*While percent negative agreement is over 90% between the two pycoMeth segmentations (Supplementary Figure 8), lower PPA on real data can likely be explained by the overabundance of low effect size DMRs.” (page 14, line 11)*

*Furthermore, we added a short Methods section (page 17, line 12) regarding how PPA is computed and interpreted and Supplementary Figure 11 illustrating it.*

* R2.3: The description of the benchmarking provided on page 11 is difficult to follow "Looking again at chromosome 20 and comparing with the tests on simulated data, only 14.8% of CpGs in DMRs found in the parent comparison are found by all segmentations, whereas in the simulated case, the agreement was 52.6%." - is the former the real data and the later the simulated dataset?

*Reply: We thank the reviewer for this question and we have rephrased the paragraph to try and make it more clear. In short, 14.8% is the agreement of all methods in the real data. 52.6% is the agreement of all methods in the simulated data. This information is also provided visually in Supplementary Figure 6. See the rewritten paragraph in response to R2.2.*

* R2.4: Figures are difficult to interpret with colours and labels not fully described. For example in figure 1 (and elsewhere), what do the orange and blue boxes indicate, in Figure 4A, what does 'no match' or 'partial match' indicate in this context - does it mean that for no DMR regions, the tool called as a DMR as a 'no match'? Some figure labels are unreadable or cut off (e.g. Figure 4B, C).

*Reply: We appreciate the comment and agree that the legends were not optimal. We updated the graphical figure legends, specifically for Figures 1, Figure 3, and Supplementary Figure 2. Specifically, the blue and orange boxes represent unmethylated and methylated methylation calls, respectively, with the color gradient representing calling certainty (the color bar representing the gradient was not rendered properly in the submission PDF, explaining why it was not visible to the reviewer).*

*The color labels in the segmentation benchmark in Figure 4A (full match, partial match, no match) are described in the figure caption of Figure 4. For the revision we slightly rephrased the description to make it more visible.*

*We have also taken care to ensure labels in Figures 4B and C which were cut off in file conversion before submission would be rendered properly in the revision.*

* R2.5: Would the authors comment on the utility of running MethylSeg/Comp on its own - or would they argue for a METEORE like approach combining calling platforms together?

*Reply: We thank the reviewer for this question, we changed this statement in the discussion section to further clarify our statement.*

*We find that MethCP and pycoMeth segmentations are very complementary, which would suggest an ensemble segmentation approach may be possible, albeit only for the purpose of downstream differential methylation calling, since the MethCP segmentation is only really meaningful in the presence of DMRs, whereas pycoMeth segmentations are meaningful in any context. Since it is hard to tell what the true biology is with regards to real segments, we imagine however, that it would be difficult to design good criteria for the combining of two segmentations.*

*As for combining DMR calls, even though every tested tool has its own strength given the run time and large overlap between the tools we would not recommend combining DMR calls from multiple calling pipelines, as it would require great care regarding multiple testing correction, which is already an issue at this large number of tests.*

We have added the following sentence to the discussion:

*“The high complementarity between changepoints placed by pycoMeth and MethCP (Supplementary Figure 6) suggest that a combined approach may be beneficial for DMR discovery. However, this does require great care in the design of the differential methylation test on which MethCP segments, and the integration of the segment boundaries, before DMR testing and multiple testing correction. Therefore we would not recommend this combination as in a general use case, and would suggest to use only pycoMeth coarse, which finds most DMR and non-DMR segments in the simulated data, as a starting point for analyses.” (page 14, line 6)*

Reviewer #3

* R3.1: The authors mentioned that the toolkit should work for GpC as well as 6mA modifications. Could they comment on the possibility of examining 5mC and 5hmC on the same CpG motifs? Is that doable in the existing implementation or will it require code development?

*Reply: Thanks for this very valuable comment. In the current version it is is not possible to examine multiple modifications at the same time.*

*However, there is a possible workaround, by modifying read id’s (such as “read_name_a_m5C” and “read_name_a_5hmC”). This is suboptimal as it requires duplicated “read_id” and “range” entries. We have ideas, however, which would allow for an easy and backward compatible way to store different modifications on the same coordinates by introducing multiple “llr” datasets which could share the same “range” and “read_id” dataset. Once implemented this will represent a very efficient storage method taking advantage of the relational design of the MetH5 format and even be a further advantage over BAM storage where read-coordinates must be repeated for each methylation call. We have added a sentence pointing at this future development at the end of our Discussion section.*

*Specifically:*

*“Although this is not currently implemented, the relational design of MetH5 also makes it an excellent choice for storing multiple nucleotide (or base) modification calls on the same data, such as for different oxidative derivatives of 5mC (5hmC, 5fC, and 5caC), by linking multiple LLR datasets to the same coordinates and read IDs.” (page 14, line 31)*

* R3.2: I appreciate the complexity and challenges associated with various file formats and incompatibilities. What is the roadmap for integrating with modBAM formats? Tools like modbamtools are handling the methylation tags now directly from Guppy basecalls which is quite nice. I think there would be an even higher adoption for pycoMeth (and possibly MetH5) with integration for that perhaps?

*Reply: We agree with this comment, which is why we provide a file conversion method in the latest development version of the MetH5 CLI which allows for conversion from BAM, with modification tags, to MetH5 format. Unfortunately, to do so we depend on pysam, which in its current version does not support the official specification of the MM tag. This is also why we used modbampy in our benchmarks (Methods), which implements a compatible reader but will likely not be supported in the future. As specified by the creators; this specific package was intended to be an ad hoc solution to bridge the gap until canonical libraries are updated. As also raised in the response to reviewer question R2.1; Directly reading BAM files in pycoMeth without conversion is something we are looking into, but early impressions are that the performance cost would be too great. Therefore, the inclusion of the converter is the best way forward, we believe.*

**Second round of review**

**Reviewer 2**

This reviewer thanks the authors for comprehensively addressing the original critiques in the revised manuscript. I have no further questions. Figure presentation could be further improved notably the extended histogram legends in figure 4B and C and supplemental figure 5.

**Authors Response**

**Point-by-point responses to the reviewers’ comments:**

Reviewer #2

This reviewer thanks the authors for comprehensively addressing the original critiques in the revised manuscript. I have no further questions. Figure presentation could be further improved notably the extended histogram legends in figure 4B and C and supplemental figure 5.

*Reply: We have modified Figure 4B and C as well the corresponding supplementary figure as requested by Referee 2. We have simplified the graphical representation to be more easily readable and elaborated in the text legend to more clearly communicate how to read these bar charts.*
